# Perspective: host factors variants and the underlying causes of long COVID

**DOI:** 10.3389/fmed.2025.1603317

**Published:** 2025-06-02

**Authors:** Tirasak Pasharawipas

**Affiliations:** Department of Medical Technology, College of Allied Health Sciences, Rattana Bundit University (Pathumthani Campus), Pathumthani, Thailand

**Keywords:** host factors variants, long COVID, cellular variants, MHC polymorphism, antigen presenting cells, long COVID diagnosis

## Abstract

Long COVID, also known as post-COVID syndrome (PCS), is characterized by persistent and unexplained symptoms that can occur not only in individuals who experienced severe symptoms during the acute phase of SARS-CoV-2 infection but also in those who were asymptomatic or had only mild symptoms. These symptoms may persist for months, even in individuals who test negative via nasopharyngeal swab samples. This proposal aims to explain the cause of long COVID through the concept of host factor variants, including cellular variants and major histocompatibility complex (MHC) polymorphisms. A key aspect of this hypothesis is the role of antigen-presenting cells, such as macrophages and dendritic cells. It is proposed that blood or white blood cells could serve as suitable samples for diagnosing long COVID in affected individuals.

## 1 Introduction

Long COVID, also referred to as post-viral sequelae or post-COVID syndrome (PCS), affects not only individuals who experienced severe symptomatic COVID-19 but also a significant number of those who were asymptomatic or only mildly symptomatic. It is estimated to affect 10–20% of SARS-CoV-2-positive individuals—and possibly more, according to some reports—even after recovery and despite negative results from nasopharyngeal swab testing (1). The condition manifests as a range of symptoms involving multiple organ systems. Common symptoms include fatigue, breathlessness, persistent cough, chest pain, difficulty speaking, muscle aches, and cognitive dysfunction, all of which can significantly impair daily activities and quality of life. These chronic symptoms may last for months or even years, depending on the individual (1, 2).

There are no predictable symptoms for diagnosing long COVID, especially in patients who are asymptomatic during the acute phase. Some individuals experience continuous symptoms during the acute phase, whereas others develop symptoms after recovering from their initial illness. Symptoms can relapse intermittently, varying from better to worse, without any clear clinical indicators of prognosis (2, 3).

SARS-CoV-2 positivity can be found in individuals who have been fully vaccinated, therefore, long COVID can also occur in some vaccinated individuals. Currently, there is no definitive explanation of the causes of long COVID (4). Surveys have indicated that age, sex, and underlying diseases are associated with its incidence; however, the true cause remains unclear and warrants further investigation (1–3).

The post-viral sequelae of other viruses have also been reported. Examples include measles (5), chikungunya (6), West Nile virus (7), and hepatitis B and C viruses, which can cause chronic infections in approximately 10–15% and 60–70% of the cases, respectively (8, 9). The occurrence of post-viral sequelae following certain acute viral infections suggests that individual host factors, rather than viral factors alone, may contribute to these outcomes. If the virus alone was responsible, it would be unclear why all exposed individuals did not develop post-viral sequelae. Variations in host factors among individuals have been proposed to explain post-viral sequelae.

The concept of host factor variants, including variations in cellular molecules and major histocompatibility complex (MHC) polymorphisms, has been proposed to explain the variable symptoms in SARS-CoV-2-positive individuals. This article expands on this concept to explain the causes of long COVID, particularly focusing on the roles of cytotoxic T cells (Tc), helper T cells (Th), and antigen-presenting cells (APCs), including details from a previous article (10).

## 2 Cellular variants and viral infection

The virus is an obligate intracellular agent that requires a susceptible host cell to enter and replicate in the host cell, therefore, it uses the receptor-binding domain (RBD) to interact with the host’s cellular molecule(s), which require susceptible receptor/co-receptor molecules for viral attachment and entry. Cellular variants have been reported in individuals susceptible to viral attachment and entry and are related to symptomatic severity (11, 12). The insusceptibility between the viral RBD and host cell variants blocks viral entry and replication in the target host cell.

Viral infection can occur only when the virus attaches to a cellular molecule with its susceptible RBD and enters the target host cell of the individual and can replicate. This is opposite to the definition of “viral expose,” which is the incidence that a virus invaded into a body, but does not enter into the target host cell because the viral RBD is not susceptible to host cellular variants. Once a virus invades the body, it does not always result in infection if the cellular variants of the individual are not susceptible to the viral RBD. This logic explains the cause of variable symptoms in SARS-CoV-2-positive individuals (10).

For most viruses, the pathogenesis is related to the host response to foreign substances, which is known as an immune-pathogenic syndrome characterized by the release of pro-inflammatory cytokines (13, 14). If individuals do not have susceptible variants of the cellular molecules that enable the virus to attach to and enter, they may be asymptomatic or mildly affected. Although the virus does not enter susceptible target cells, it can enter APCs, such as macrophages and dendritic cells (DCs) with their broadly acceptable receptors to foreign substances, known as toll-like receptors (TLRs) (15). During this period, virus-exposed individuals may show symptoms owing to the release of pro-inflammatory cytokines by APCs. APCs present viral epitopes that induce adaptive immune cells, such as Tc and Th cells in secondary lymphoid organs (13, 16).

During viral presentation by APCs, virus-exposed individuals may show symptoms due to the effects of pro-inflammatory cytokines (17). This situation differs from a viral infection, in which the virus enters the target cells and extensively replicates. In individuals genetically susceptible to viral infections, the target cell becomes a source for the virus to multiply, leading to an uncontrolled release of inflammatory cytokines and severe symptoms (18). The discussion of long COVID and the critical roles of MHC polymorphisms in individuals will be explored in more detail later.

## 3 The relationship between the polymorphism of MHC molecules and the individuals immune responses

MHC molecules are located in the cell membrane. There are two classes of MHC molecules, I and II. MHC class I molecules are found on the membranes of all nucleated cells in the body. In contrast, MHC class II molecules are expressed only on the membranes of APCs to activate Th cells. Each class of MHC molecules has multiple loci that are recognized as classical and non-classical. In humans, MHC molecules are called human leukocyte antigens (HLAs), because they were first studied and discovered in white blood cells. Therefore, HLA is synonymous with human MHC molecules. Classical HLA class I loci include HLA-A, HLA-B, and HLA-C, whereas HLA class II loci include HLA-DP, HLA-DQ, and HLA-DR.

HLA molecules are inherited from both parents, which indicates that each locus in an individual’s HLA genome can be heterozygous or homozygous. Consequently, the number of gene alleles for each MHC class in any individual was limited to three to six. For example, an individual homozygous at all three loci would have only three gene alleles, whereas an individual heterozygous at all loci would have six gene alleles. MHC gene alleles are highly polymorphic, the likelihood that two individuals will have the same set of gene alleles is <1 in a million and is most commonly observed in identical twins.

Individuals who are MHC homozygous are more susceptible to pathogens than those who are MHC heterozygous (19, 20). To explain this more extensively, the interaction between MHC molecules and T cell epitopes in the formation of pMHC complexes to activate T cell clones is crucial. Normally, MHC molecules do not need to interact with all the amino acids of a T-cell epitope, which typically consists of approximately 8–20 amino acid residues. MHC molecules require only a few amino acids, approximately 2–4 residues, of the T-cell epitope for interaction to form a pMHC complex (21, 22). The region of the T cell epitope that interacts with the MHC groove is referred to as the anchor residue. This allows each MHC allele to bind to many different peptides, provided that the anchor residues are present in specific T cell epitopes. Thus, any T cell epitope peptide processed by APCs must contain amino acids that can serve as anchor residues and fit into the MHC allele pocket to form a pMHC complex (22, 23). The formation of pMHC is pivotal for inducing T-cell clones via the T-cell receptor (TCR), although many other molecules are involved in the interaction between T cells and APCs (24). Each MHC variant can bind to many different peptides, giving MHC molecules broad interactions with T cell epitopes present in APCs. However, each MHC allele has limitations in binding to certain peptides (25–27). It is unlikely that all processed T cell epitopes can form a pMHC complex with a single MHC allele.

Thus, the MHC alleles of each individual are limited in forming pMHC complexes with some peptides if the T-cell epitopes do not contain anchor residues compatible with the individual’s MHC alleles. This explains why individuals with fewer MHC alleles are reportedly more susceptible to pathogenic infections. This could be due to the limitations in forming pMHC complexes with foreign substances, including viral agents, to induce naïve T-cell clones in these individuals. In addition to the possibility of viral variants, the lack of compatible MHC alleles in individuals may explain why some individuals become infected and do not respond efficiently to gain seroprotection even after receiving full doses of the viral vaccine (28, 29).

Therefore, the invasion and infection of any particular foreign substance, including viral antigens, in different individuals results in varying levels of immune response. To activate Tc cells, individuals require a compatible MHC class I allele to form a pMHC-I complex with the viral epitope. Similarly, a compatible MHC class II allele is required to form the pMHC-II complex to activate Th cells.

## 4 The role of macrophages and DCs as APCs

The adaptive immune response requires that macrophages and DCs present antigenic epitopes for primary activation. Both macrophages and DCs function as APCs because of their ability to express MHC class I and II, forming pMHC-I and pMHC-II complexes for T-cell clone activation. Although DCs may play a crucial role in activating T-cell clones (30), macrophages are more active in removing apoptotic cells and foreign agents through phagocytosis (31). While acting as APCs, both macrophages and DCs produce cytokines as mediators that initiate antigen presentation.

APCs, macrophages, and DCs capture foreign substances, including viral agents, through TLRs (15). This process is essential; antigen presentation cannot occur without antigen engulfing, and T cell activation becomes impossible. There is no direct evidence of the pathogenesis of most viruses. Instead, macrophages and DCs, which function as APCs, are the primary sources of pro-inflammatory cytokines that can lead to mild-to-severe symptoms in response to the viral presence in the body.

In virus-infected individuals, viral replication occurs within susceptible target cells. In contrast, in individuals exposed to viruses, viral agents are present only in limited amounts and are engulfed by APCs. Some studies have identified viral agents within APCs, particularly macrophages and DCs, suggesting that certain viruses target these cells (32–38). However, it is important to consider that this may be part of the APC process rather than evidence that APCs are the direct target cells for these viruses. Further investigation is needed to clarify this for each viral agent identified in APCs. The persistent presence of viral agents in APCs has been observed in COVID 19 (32, 33), including respiratory syncytial (34), hepatitis B (35), and hepatitis C viruses (36), and many others (33, 37, 38). Previous studies have mainly attributed viral persistence in APCs to the immune evasion mechanisms employed by these viruses. However, it remains unclear why immune evasion occurs in some individuals, but not in others and why the prevalence varies among different viruses. These differences may be explained by variations in host factors or potentially by a combination of viral and host factors influenced by individual genetics. Further details of these mechanisms are discussed later.

There are two types of macrophages: M1 and M2. M1 macrophages primarily produce proinflammatory cytokines, whereas M2 macrophages produce anti-inflammatory cytokines. However, both M1 and M2 macrophages can switch between phenotypes with one another (39). DCs also have heterogeneous forms and produce various pro-and anti-inflammatory cytokines. Currently, DCs are classified into four distinct subpopulations: conventional (cDC1 and cDC2), plasmacytoid (pDCs), and monocyte-derived (mo-DCs) DCs (36, 37). cDC1 is rare in the body, whereas cDC2 is abundant. Further studies are required to understand the phenotypic diversity and specific functions of these heterogeneous DC phenotypes, although cytokine production and molecular expression have been described for each phenotype (40).

Notably, macrophages and DCs are both derived from the same progenitor and capable of switching functions with one another. Macrophages perform DC functions to a certain extent, and vice versa (41). However, there is no conclusive evidence that these two cells are identical. This contrasts with monocytes and macrophages, which have been classified as the same type of cells after decades of research. A key question arises: Why does the immune system require both macrophages and DCs to play similar roles as APCs if they are distinct cell populations? This raises challenges for reviewing and integrating knowledge concerning the mediating role of APCs in linking innate and adaptive immunity. Further studies in this area may provide clearer and more conclusive results.

Based on fundamental knowledge, it was concluded that macrophages and DCs are APCs that play a central role in producing pro-inflammatory cytokines during their functions. Cytokine production probably contributes to the pathogenesis of viral invasion and infection. Additionally, there is no evidence that macrophages or DCs can clear viral agents by themselves, effective Tc cells are required for this process, which will be discussed later.

## 5 Host factor variants concept for explaining the cause of long COVID in asymptomatic SARS-CoV-2-positive individuals

The *host factor* var*iants concept* has been proposed to classify individuals into eight groups based on three host factors: (1) viral-susceptible variants of cellular molecules, (2) compatible MHC class I alleles, and (3) compatible MHC class II alleles. This framework helps explain the variable symptoms observed in individuals after viral invasion. Individuals without susceptible variants of the cellular molecules were not truly infected. Only patients with susceptible cellular variants experienced severe symptoms. For viral clearance, individuals must possess MHC class I compatibility to activate Tc cells, which require compatible Th cells for further induction into effective Tc and memory Tc cells, including memory B cells. Compatible Th cells require MHC class II compatibility to present the viral epitope.

Table 1 explains the cause of long COVID in asymptomatic or mildly symptomatic SARS-CoV-2-positive individuals based on the host factor variant concept. These individuals remain asymptomatic or have mild symptoms because they lack susceptible cellular variants for the viral RBD to attach to and enter host cells. Thus, the virus invades the body without replicating in its primary target cells, thereby preventing complete infection. Individuals in Group 1 were predicted to be the least affected, as viral invasion may cause only minor symptoms or leave them asymptomatic while still allowing for a full adaptive immune response to clear the virus and provide protection. They may remain healthy even after exposure to the virus without vaccination.

**Table 1:** Classification of individuals who are insusceptible to viral infection and became symptomless/mild symptoms of SARS-CoV-2 invasion while the compatibility of their MHC alleles determines the potential to clear the viral agent from the viral engulfing APCs, which are persistent in becoming the cause of long COVID or post COVID syndrome (PCS).

Individual group	MHC I allele	MHC II allele	Prediction on viral exposure and the possibility to gain post COVID syndrome (PCS)
1	Compatible	Compatible	With the complete production of an effective Tc and Th cell clones and all of the memory cells of adaptive immune cells, it is unlikely that these individuals will gain PCS
2	Compatible	Non-compatible	With specific Tc cells that cannot develop into effective T cells due to the lack of effective Th cell, only IgM is produced, and no memory B and T cells are formed. PCS is possible.
3	Non-compatible	Compatible	Although effective Th cells and memory B cells are generated, along with all classes of immunoglobulins, the lack of specific Tc cells allows for the possibility of PCS
4	Non-compatible	Non-compatible	Without the generation of both effective Tc and Th cell, PCS is highly predictable in this group.

In contrast, individuals in Groups 2–4 had one or both classes of MHC incompatible with viral peptides, preventing the formation of specific pMHC complexes required to induce effective T-cell clones. This incompatibility may lead to T-cell exhaustion, impairing the ability of the body to clear virus-infected cells (42). This mechanism may also help explain the cause of long COVID in some individuals who retain the viral agent in APCs, rather than attributing it solely to immune evasion by the virus. Viral mutations in hotspot regions can alter the amino acids of a T-cell epitope, changing its compatibility with certain MHC alleles and leading to incompatibility, or vice versa. Consequently, individuals who are unable to form compatible pMHC complexes to activate T-cell receptors of specific T-cell clones may develop chronic infections or post-viral sequelae. However, this hypothesis requires further investigation.

Notably, although SARS-CoV-2 transcription and translation have been observed in monocytes/macrophages, infectious viral particles have not been detected (43). This suggests that the virus undergoes abortive infection within antigen-presenting cells (APCs). The specific mechanisms underlying this phenomenon remain unclear and may involve disruptions at various stages of the viral replication process. Similar patterns have been observed with other viruses. For example, in the case of influenza virus, impairments in viral assembly or budding within APCs have been reported (44, 45). Abortive infections in APCs have also been documented for other viruses (46, 47). Further studies are needed to elucidate both the mechanisms that block viral production and the underlying causes of abortive infections in APCs. Nevertheless, it is arguably beneficial that APCs are not ideal targets for productive viral infection. Their primary role is to activate T-cell clones through antigen presentation; if they were permissive to viral replication, they could instead become centers of viral proliferation and excessive proinflammatory cytokine production, potentially triggering harmful cytokine storms in larger segments of the population, including individuals merely exposed to the virus but not actively infected.

Despite the inability of SARS-CoV-2 and other viruses to complete their replication cycles in APCs, the induction of inflammatory cytokines can still occur, as demonstrated in previous studies (48, 49). Notably, the persistence of spike protein in non-productively infected APCs has been associated with sustained cytokine production, potentially contributing to long COVID symptoms (43). In addition, the study by Zheng et al. (48) reported that abortive infection of SARS-CoV-2 in macrophages can induce a strong inflammatory response via activation of Toll-like receptors (TLRs) and inflammasomes. These findings may help explain how abortive infection of APCs by SARS-CoV-2 contributes to inflammatory cytokine production in long COVID patients.

Considering the antigen-presenting roles of macrophages and DCs in individuals in Groups 2–4, these APCs were suspected of harboring the viral agent. Macrophages and DCs can migrate and disseminate the viral agent to various organs, acting as sources of pro-inflammatory cytokines that contribute to persistent symptoms associated with long COVID (50, 51). As an immune-privileged organ, the central nervous system (CNS) might be accessed by APCs carrying the virus across the blood–brain barrier via a “Trojan horse” mechanism, causing inflammation and viral CNS infection in patients with long COVID (52, 53). Additionally, there have been reports of persistent viruses in macrophages and DCs during chronic viral infection (54, 55). The discovery of viral particles in APCs, based on autopsies of individuals with chronic viral symptoms, has been previously reported (56, 57). These studies could provide key insights for understanding the causes of post-viral sequelae in various viral infections, including long COVID. From an immunological perspective, macrophages and DCs are not capable of clearing viral agents on their own, they rely on effective Tc cells. It should be evaluated whether the virus within APCs is part of their duty to present antigenic epitopes to activate adaptive immune cells, rather than simply being the result of viral infection. The T cell epitopes of viral agents require compatible MHC alleles to form appropriate pMHC complexes that activate the TCR of either Tc cells through MHC class I alleles or Th cells through MHC class II alleles. Incompatibility in forming either or both pMHC-I and pMHC-II complexes halts further activation of adaptive cellular immunity, preventing the activation of T-cell clones. This can result in persistent viral agents in the macrophages and DCs, contributing to post-viral sequelae. Such a mechanism might explain T cell exhaustion, in which T cells lose their ability to eliminate infected cells, resulting in chronic infection or post-viral sequelae in virus-invaded individuals.

Based on this perspective, SARS-CoV-2-engulfing macrophages and DCs may contribute to long COVID in individuals from Groups 2–4 (Table 1). The incompatibility of viral peptides and MHC alleles in these individuals suggests that those in Group 4 might experience more severe and prolonged symptoms than those in Groups 2 and 3 because they are unable to generate effective Tc and Th cells. Conversely, predicting the outcomes of the individuals in Group 2 is challenging. Whether activated Tc cells, which do not further develop into effective Tc cells due to a lack of Th cell assistance, can clear viral-engulfing APCs remains unclear (58–60). Effective T-cell generation requires tripartite interactions between APCs, Tc cells, and Th cells (61). This step is crucial for clearing virus-engulfing APCs and preventing the persistence of the virus, which can then disseminate to various organs. Further studies are required to elucidate these mechanisms.

For individuals in Group 3, who lack compatible Tc cells, symptoms may resemble those of Group 4 unless natural killer cells, in combination with IgG antibodies through antibody-dependent cytotoxicity, clear the viral agent (/). This presents a notable avenue for research, particularly for organizations with the potential to conduct clinical studies on HLA alleles in patients with long COVID.

## 6 Peripheral blood analysis for long COVID diagnosis

Peripheral blood samples can be analyzed for the SARS-CoV-2 genome using reverse transcription PCR (RT-PCR) (62), in conjunction with cytokine level assessments, to aid in the diagnosis of long COVID. Although SARS-CoV-2 has been reported to undergo abortive infection in APCs, it can still induce inflammatory cytokine production. This is likely due to continued viral peptide synthesis, which may act as a trigger for the inflammatory response contributing to long COVID symptoms (43, 48, 49). Given that the SARS-CoV-2 genome can persist in long COVID patients, APCs may serve as a potential viral reservoir.

Analyzing peripheral blood or white blood cells for SARS-CoV-2 genome detection may yield valuable diagnostic markers, enabling a faster and more specific diagnosis of long COVID compared to cytokine profiling alone. Early detection in individuals who test negative by nasopharyngeal swab could help confirm viral persistence, monitor disease progression, and inform preventive strategies. This approach may also provide insight into post-viral sequelae from other viral infections, such as dengue (63), influenza (64), and others (32–37). Additionally, recent findings linking iron dysregulation and inflammatory stress erythropoiesis to post-COVID syndrome (PCS) suggest another potential diagnostic pathway (65). Early diagnosis could support preventive interventions—such as iron supplementation—to reduce the risk of long COVID. Research into iron metabolism may also be applicable to other post-viral conditions.

The clinical implications of this research include the development of early diagnostic tests for long COVID, particularly in patients who are PCR-negative via nasopharyngeal swabs yet continue to experience persistent symptoms. Identifying individuals at risk based on cytokine profiles and iron metabolism abnormalities could allow for timely therapeutic interventions, including iron supplementation, to mitigate long-term sequelae. Future studies may investigate whether antiviral or immunomodulatory therapies can reduce viral persistence. Multi-center studies are warranted to validate potential biomarkers and explore the autoimmune components potentially associated with long COVID. By integrating molecular diagnostics, immune profiling, and iron metabolism analysis, this approach aims to improve the management of long COVID and guide targeted therapeutic strategies.

## 7 Conclusion

This study proposes that the long COVID in asymptomatic or mildly symptomatic SARS-CoV-2-positive individuals can be explained by the host factor variant concept. These individuals are exposed by SARS-CoV-2 without substantial replication in target cells. Individuals with incompatible MHC alleles for both classes I and II (Group 4), who cannot generate effective Tc and Th cells, are more likely to develop long COVID. Patients with incompatible MHC class I or class II alleles (Groups 2 and 3) may also be at risk. Impaired T-cell function in these individuals may allow the virus to persist in APCs, which could be a key cause of persistent symptoms due to the release of pro-inflammatory cytokines. Persistent viral agents in APCs may migrate and disseminate to various organs, including the CNS, leading to persistent symptoms. This study also suggests that examining the SARS-CoV-2 genome in peripheral blood or white blood cells could provide a diagnostic tool for long COVID, facilitating early diagnosis and better management of the condition. However, this approach requires further investigation.

## Data Availability

The raw data supporting the conclusions of this article will be made available by the authors, without undue reservation.
